# Changes in and the mediating role of physical activity in relation to active school transport, fitness and adiposity among Spanish youth: the UP&DOWN longitudinal study

**DOI:** 10.1186/s12966-020-00940-9

**Published:** 2020-03-10

**Authors:** Daniel Camiletti-Moirón, Anna Timperio, Jenny Veitch, Jorge Del Rosario Fernández-Santos, Gavin Abbott, Álvaro Delgado-Alfonso, Veronica Cabanas-Sanchez, Oscar L. Veiga, Jo Salmon, José Castro-Piñero

**Affiliations:** 1 Department of Physical Education, GALENO Research Group, School of Education Sciences, University of Cádiz, Avenida República Saharaui s/n, 11519, Puerto Real, Cádiz, Spain; 2 Biomedical Research and Innovation Institute of Cádiz (INiBICA) Research Unit, Puerta del Mar University Hospital University of Cádiz, Cádiz, Spain; 3 Institute for Physical Activity and Nutrition (IPAN), School of Exercise and Nutrition Sciences, Deakin University, Geelong, Australia; 4 Department of Physical Education, Sports, and Human Movement, Autonomous University of Madrid, Madrid, Spain

**Keywords:** Active commuting, Obesity, Fitness, Childhood, Adolescence

## Abstract

**Background:**

Longitudinal changes in child and adolescent active school transport (AST), and the mediating role of different intensities of daily physical activity (PA) levels in relation to AST and physical fitness and adiposity indicators is unclear. This study aimed to: 1) describe longitudinal changes in AST, light PA (LPA), moderate- to vigorous-intensity PA (MVPA), physical fitness and adiposity indicators over three time-points; and 2) investigate the mediating role of LPA and MVPA levels on associations between AST and physical fitness and adiposity indicators over three time-points among children and adolescents.

**Methods:**

This longitudinal study comprised 1646 Spanish children and adolescents (48.8% girls, mean age 12.5 years ±2.5) at baseline, recruited from schools in Cádiz and Madrid. Mode of commuting to school was self-reported at baseline (T0, 2011–12), 1-year (T1) and 2-year follow-up (T2). PA was assessed using accelerometers. Handgrip strength, standing long jump and cardiorespiratory fitness (CRF) assessed physical fitness. Height, weight, body mass index, waist circumference, and triceps and subscapular skinfold thickness were measured. Multilevel linear regression analyses assessed changes in AST, PA levels, physical fitness and adiposity indicators over three time-points (T0-T1-T2). Additionally, longitudinal path analysis (*n* = 453; mean age [years] 12.6 ± 2.4) was used to test the mediating effects of LPA and MVPA levels on the association between AST and physical fitness and adiposity indicators.

**Results:**

Multilevel analyses observed decreases in LPA between T0-T1 (β = − 11.27; *p* < 0.001) and T0-T2 (β = − 16.27; *p* < 0.001) and decreases in MVPA between T0-T2 (β = − 4.51; *p* = 0.011). Moreover, changes over time showed increases in handgrip between T0-T1 (β = 0.78; *p* = 0.028) and T0-T2 (β = 0.81; *p* = 0.046).

Path analyses showed that AST was directly positively associated with MVPA at T1 (all, β ≈ 0.33; *p* < 0.001). MVPA at T1 mediated associations between AST and CRF at T2 (β = 0.20; *p* = 0.040), but not the other outcomes. LPA did not mediate any associations.

**Conclusions:**

Results from longitudinal path analysis suggest that participation in more AST may help attenuate declines in MVPA that typically occur with age and improve CRF. Therefore, we encourage health authorities to promote AST, as a way to increase MVPA levels and CRF among youth.

## Background

The lack of physical activity (PA) is a global public health problem, responsible for more than five million deaths annually through its effects on multiple non-communicable diseases across the population [1–3]. In response to this, international health authorities [4–7] have adopted policy strategies to incorporate PA into everyday life to reduce the growing global burden of chronic diseases [8]. Despite international recommendations for children and adolescents to accumulate at least 60 min of moderate- to vigorous-intensity physical activity (MVPA) daily [9], however, more than half of children and adolescents worldwide do not meet these guidelines [10, 11].

Active transport, mainly walking and cycling, has been recommended as a consistent and practical way to integrate more PA into daily life [12]. In addition, active transport, principally cycling, has been associated with a lower risk of cardiovascular disease (CVD), cancer, and all-cause mortality in adults [13–15]. Active school transport (AST) provides an important opportunity for children and adolescents to increase daily PA levels [16–18]. Moreover, there is consistent cross-sectional and longitudinal evidence that AST is associated with greater cardiorespiratory fitness (CRF), mainly when AST is by bicycle [19–21] and muscle strength of the lower body is involved [21–23]. AST may also have considerable potential to reduce obesity in young populations [24]. However, there is substantial scientific uncertainty concerning the nature and strength of associations between different modes of travel, such as AST, and adiposity [20, 25]. Longitudinal studies with more than two time-points that have examined associations between AST and PA levels, physical fitness and adiposity indicators are scarce [8, 19]. Light PA (LPA) is a recent target in 24 h movement guidelines. Literature shows the potential benefits of LPA for CRF and adiposity indicators [26] and the increasing interest to reallocate sedentary time, which is detrimental for health, to LPA time, once adequate time in sleep and MVPA have been performed [27]. However, few studies have reported on LPA changes over time or the role of this intensity in relation to key PA behaviors such as AST [26, 28]. This provides a timely opportunity to explore PA beyond MVPA.

Consequently, the aims of this study were: 1) to describe longitudinal changes in AST, LPA, MVPA, physical fitness and adiposity indicators over three time-points; and 2) to investigate the mediating role of LPA and MVPA levels on associations between AST and physical fitness and adiposity indicators over three time-points among children and adolescents.

## Methods

### Participants and study design

Participants were recruited as part of a larger multi-centre longitudinal study, UP&DOWN [29]. The UP&DOWN study included a sample of 2225 healthy children (6–11.9 years) and adolescents (12–17.9 years) recruited from schools with Principal consent in Cádiz and Madrid, respectively (i.e. Primary Schools in Cádiz, *n* = 23 and Secondary Schools in Madrid, *n* = 22). According to the database of the Spanish Institute Statistics, our recruited sample represented 50% of the total Cádiz primary school children (*n* = 1188) and 5% of the total Madrid secondary school adolescents (*n* = 1037), respectively. Baseline data collection occurred from September 2011 to end June 2012 (T0). Participants were followed up 1-year (T1) and 2-years (T2) later. For the current analysis, 6- and 7-year-old children were not included (*n* = 579), because they were not able to complete self-reported measures regarding the mode and frequency of AST of a valid questionnaire [30]. Furthermore, those youth who did not provide complete data at T0, T1 and T2 on AST volume, PA levels, physical fitness and adiposity indicators were excluded (*n* = 1193). Thus, the final sample included 453 youth (218 girls) aged 8.9–17.9 years (mean, 12.6 ± 2.4 years). This study followed the ethical standards recognized by the Declaration of Helsinki; the study protocols were approved by the Ethics Committee of the Hospital Puerta de Hierro (Madrid, Spain; case number 269, 26/09/2011), the Bioethics Committee of the CSIC and the Ethics Committee for Research Involving Human Subjects at UCA (Cádiz, Spain; 13/10/2011). The study was explained to the participants before starting, and parents or tutors provided informed consent.

### Assessment of mode and frequency of commuting to and from school

To assess the mode and frequency of commuting to and from school, a validated self-reported Spanish questionnaire was used [30]. Participants completed the questionnaire in the classroom. The questionnaire included six questions: (1) usual mode of commuting to school, (2) usual mode of commuting from school, (3) usual weekly number of school trips by mode of commuting to school, (4) weekly number of school trips by mode of commuting from school, (5) time taken to travel actively from home to school and (6) time taken to travel actively from school to home during weekdays. Modes of commuting included: walk, cycle, car, motorcycle, bus or other (specified by the respondent). A binary (active/passive) variable was obtained from the questions about usual mode of commuting to and from school, which was only used for descriptive data. The use of car, motorcycle or bus were categorized as passive, and walking and cycling (*n* = 8) were categorized as active. Those who were usually passive on both trips to and from school were categorized as passive participants; those who usually active on at least one way (to or from school) were categorized as active participants. Additionally, the total week volume of AST was calculated by summing the minutes of active trips on their way to and from school.

### Physical activity

We used the GT1M, GT3X and GT3X^+^ accelerometers (Actigraph, Pensacola, Florida, USA) to measure activity counts. These different models have been shown to provide comparable data [31, 32]. Participants wore the device on their lower back, underneath clothing and secured with an elastic belt [33]. They received instructions to remove the accelerometer during sleep and water-based activities. PA was recorded for up to seven consecutive days. A total of 3 days with a minimum of 10 valid hours per day was the inclusion criteria [34]. Non-wear time was defined as a period of 60 min of zero counts and an allowance of up to two consecutive minutes of < 100 counts per minute (cpm) [34]. Before analyses, we reintegrated data into 10-s epochs [34]. LPA, moderate, vigorous and MVPA intensity levels were calculated based upon recommended PA vector magnitude cut points [35]: 100–1999, 2000–3999, ≥4000 and ≥ 2000 cpm, respectively, and were expressed as minutes per day. We used the manufacturer software (Actilife v.6.6.2 desktop) to download, clean and analyze data.

### Physical fitness

The 20-m shuttle run test was used to assess CRF according to the Assessing Levels of Physical Activity (ALPHA) health-related fitness test battery protocol [36]. The 20-m shuttle run test is highly valid [37] and reliable [38] for assessing CRF in youth. The equation reported by Leger et al. [39] was used to estimate maximum oxygen consumption. A hand dynamometer with an adjustable grip (TKK 5101 Grip D; Takey, Tokyo, Japan) was used to measure upper body strength (handgrip). The test was performed twice, and the maximum score for each hand was recorded in kilograms. Subsequently, the average score of the left and right hands combined was calculated. The standing long jump test was carried out to measure lower body strength. Participants repeated the test twice and the longer distance was recorded in centimetres.

### Assessment of adiposity indicators

Weight was measured with an electronic scale (Type SECA 861; range, 0.05–130 kg; precision, 0.05 kg), height (measured in the Frankfort plane) with a telescopic stature-measuring instrument (Type SECA 225; range, 60–200 cm; precision, 1 mm) and waist circumference with a non-elastic tape (SECA 200; range, 0–150 cm; precision, 1 mm). We calculated body mass index (BMI) as weight/height squared (kg/m^2^). Skinfold thicknesses (mm) were measured at the triceps and subscapular on the non-dominant side of the body using a Holtain caliper (range, 0–40 mm; precision, 0.2 mm) according to the Lohman et al. [40] anthropometric standardization reference manual. We performed the measurements twice, and the mean value of the two measurements was used in the analyses.

### Socioeconomic status

The Family Affluence Scale (FAS) is based on the concept of material conditions in the family to base the selection of items [41]. Thus, FAS questionnaire was used in the present study as an index of socioeconomic status (SES) [42], which includes 4 questions answered by the participant: (1) Do you have your own bedroom?, (2) How many cars are there in your family?, (3) How many computers are there in your home?, and (4) During the past 12 months, how many times did you travel away on holiday with your family? We defined low, medium and high SES based on the final score obtained from the four questions. That is, we gave a numerical value to each possible answer in the four questions. Then we summed the final score from all the questions being ranged from 0 to 9. Finally, we grouped these scores in three levels: low (from 0 to 3), medium (from 4 to 6) and high (from 7 to 9).

### Statistical analyses

Descriptive data are presented as means and standard deviations for continuous variables and percentages for categorical variables. Differences in baseline characteristics (age, children/adolescents, sex, urban/rural status, SES, LPA and MVPA, adiposity indicators, physical fitness, mode of commuting and total volume of AST) between participants with complete data (*n* = 453) and those excluded from analysis due to missing data (*n* = 1193) were tested using t-tests for continuous variables and chi-squared tests for categorical variables.

**Fig. 1:**
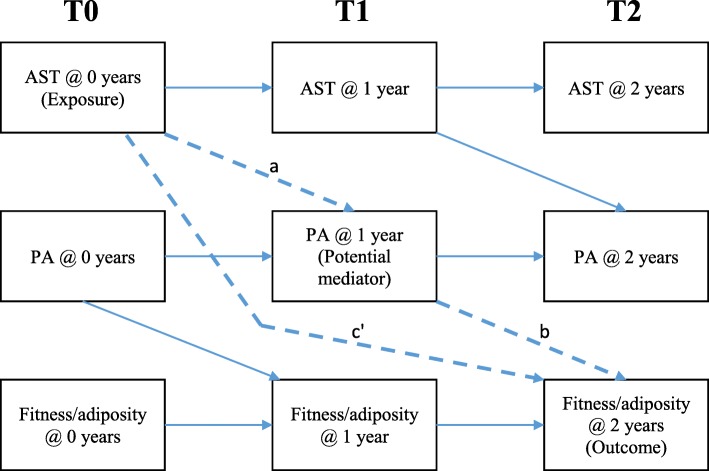
Mediation model illustrating all direct pathways and mediation pathways (dashed line). Abbreviations: AST, Active School Transport; PA, Physical Activity; T0, baseline; T1, 1-year follow-up; T2, 2-year follow-up. Path *a*, the effect of the AST at T0 (exposure) on PA at T1 (potential mediator), adjusted for T0 level of PA; Path *b*, the effect of PA at T1 on the outcome at T2, adjusted for level of the outcome at T1; Path *c’,* the direct effect of T0 AST on the outcome at T2, adjusted for T1 levels of both the outcome and PA

Linear mixed models with random intercepts for individuals were fitted to examine whether total volume of AST, LPA, MVPA, physical fitness and adiposity indicators changed over time (T0-T1-T2). Path analysis was used to model the longitudinal relationships between AST (exposure), PA (LPA or MVPA; potential mediators), and the outcomes of physical fitness and adiposity indicators across the three time-points (*n* = 453). Separate path models were tested for each PA/outcome variable pairing. Direct pathways between AST, PA, and fitness/adiposity outcome variables are shown in Fig. 1. The models also included covariance between measures taken at the same time-point. Effects dashed in Fig. 1 show the following hypothesised mediating pathways: the effect of the AST at baseline (exposure) on PA at T1 (potential mediator), adjusted for the baseline level of PA (path *a*); the effect of PA at T1 on the outcome at T2, adjusted for level of the outcome at T1 (path *b*); and the effect of baseline AST on the outcome at T2, adjusted for the T1 levels of both the outcome and PA (the ‘direct’ effect, path *c* ’).

For each model, the indirect effect of baseline AST on the outcome at 2 years occurring via the potential mediator (year 1 PA), equal to the product of paths *a* and *b*, was calculated and confidence intervals produced to allow inference. Bias-corrected bootstrapping with 1000 resamples was used to produce standard errors and 95% confidence intervals for all estimated effects in the models. All analyses were adjusted by sex, age and SES; robust standard errors were used to account for potential clustering within the 23 primary schools and the 18 secondary schools. Statistical significance was set at *p* < 0.05 and the analyses were conducted using Stata/SE 14 (StataCorp, TX).

## Results

Baseline characteristics of participants in the whole sample and for complete cases are shown in Table 1**.** Changes over time in PA levels, physical fitness, adiposity indicators and AST for complete cases over three time-points (T0-T1-T2) adjusted for sex, age and SES are presented in Table 2. Decreases in LPA were observed between T0-T1 (β = − 11.27; *p* < 0.001) and T0-T2 (β = − 16.27; *p* < 0.001) and decreases in MVPA between T0-T2 (β = − 4.51; *p* = 0.011). Increases in handgrip were observed between T0-T1 (β = 0.78; *p* = 0.028) and T0-T2 (β = 0.81; *p* = 0.046). No significant changes in AST, or other measures of physical fitness or indicators of adiposity were observed (all, *p* > 0.05). Results for the whole sample are presented in Supplementary Table 1.

**Table 1:** Demographic characteristics at baseline

	Whole sample (***n*** = 1646)	Complete case (***n*** = 453)	Excluded cases (***n*** = 1193)	***P*** value
Age, mean (SD)	12.5 (2.5)	12.6 (2.4)	12.4 (2.4)	0.224
**Age group**, **n (%)**				
Children	611 (37.1)	137 (30.2)	474 (39.7)	**< 0.001**
Adolescents	1035 (62.9)	316 (69.8)	719 (60.3)	
**Sex**, **n (%)**				
Boys	843 (51.2)	235 (51.9)	608 (51.0)	0.741
Girls	803 (48.8)	218 (48.1)	585 (49.0)	
**Urban/rural**, **n (%)**				
Urban	789 (47.9)	235 (51.9)	554 (46.4)	**0.049**
Rural	857 (52.1)	218 (48.1)	639 (53.6)	
**Socioeconomic Status** (*n* = 1586)^a^, **n (%)**				
Low	173 (10.9)	38 (8.4)	135 (11.9)	**0.040**
Medium	639 (40.3)	173 (38.4)	466 41.0)	
High	774 (48.8)	239 (53.1)	535 (47.1)	
**Physical Activity Levels** (*n* = 1445)^a^, mean (SD)				
LPA (min/day)	178.8 (46.5)	177.5 (44.4)	179.3 (47.4)	0.489
MVPA (min/day)	68.7 (25.6)	66.3 (25.0)	69.8 (25.7)	**0.014**
**MVPA Recommendations** (*n* = 1445)^a^, **n (%)**				
≥ 60 min/day	853 (59.0)	240 (53.0)	613 (61.8)	**0.002**
< 59.9 min/day	592 (41.0)	213 (47.0)	379 (38.2)	
**Adiposity indicators** (*n* = 1616)^a^, mean (SD)				
Weight (kg)	48.2 (14.2)	48.8 (13.9)	48.0 (14.3)	0.293
Height (cm)	152.3 (13.9)	153.8 (13.4)	151.7 (14.0)	**0.007**
BMI (kg/cm^2^)	20.4 (3.6)	20.2 (3.4)	20.4 (3.7)	0.371
Waist circumference (cm)	66.4 (8.7)	66.3 (8.3)	66.5 (8.8)	0.788
Skinfolds (Sum of triceps and subscapular) (*n* = 1610)^a^	27.4 (13.3)	26.7 (12.3)	27.6 (13.7)	0.218
**Physical Fitness**, mean (SD)				
Standing long jump (cm) (*n* = 1609)^a^	145.1 (33.2)	147.3 (32.5)	144.3 (33.4)	0.098
Handgrip strength (kg) (*n* = 1611)^a^	21.7 (8.3)	22.0 (8.0)	21.6 (8.5)	0.322
20-m shuttle run test (CRF) (VO_2max_ ml/kg/min) (*n* = 1568)^a^	33.3 (7.9)	33.8 (8.2)	33.2 (7.8)	0.183
**Mode of commuting** (*n* = 1530)^a^				
Active, n (%)	1017 (66.5)	237 (53.1)	780 (72.0)	**< 0.001**
Passive, n (%)	513 (33.5)	209 (46.9)	304 (28.0)	
**Change AST T0-T2** (*n* = 1233)^a^, **n (%)**				
Active Persistent	724 (58.7)	227 (50.6)	497 (63.4)	**< 0.001**
Passive Persistent	348 (28.2)	184 (41.0)	164 (21.0)	
Active T0 - Passive T2	75 (6.1)	13 (2.9)	62 (7.9)	
Passive T0 - Active T2	86 (7.0)	25 (5.6)	61 (7.8)	
AST volume (min/week) (*n* = 1254)^a^, mean (SD)	86.0 (95.0)	76.3 (93.7)	91.5 (95.4)	**0.007**

Abbreviations: *SD* Standard Deviation, *LPA* Light Physical Activity, *MVPA* Moderate-to-Vigorous Physical Activity, *BMI* Body Mass Index, *CRF* Cardiorespiratory fitness, *VO*_*2max*_ Maximum Oxygen Consumption, *T0* Baseline, *T2* 2-year follow-up, *AST* Active School Transport. ^a^Whole sample reduced due to missing values from youth who did not provide complete data at T0, T1 and T2 on AST volume, PA levels, physical fitness and adiposity indicators. *P value* shows differences between complete case and excluded case

**Table 2:** Changes over time in AST volume, PA, adiposity and fitness for complete case (*n* = 453)

	1-year follow-up			2-year follow-up		
	β	95%CI	***P*** value	β	95%CI	***P*** value
**Mode of commuting**						
AST volume (min/week)	8.18	−4.50, 20.86	0.206	6.89	−7.59, 21.36	0.351
**Physical Activity Levels**						
LPA (min/day)	−11.27	− 15.79, −6.76	**< 0.001**	− 16.27	− 21.42, − 11.12	**< 0.001**
MVPA (min/day)	−1.86	− 4.91, 1.20	0.233	− 4.51	−7.99, − 1.03	**0.011**
**Adiposity indicators**						
BMI (kg/cm^2^)	0.18	− 0.26, 0.61	0.430	− 0.10	− 0.60, 0.40	0.700
Skinfolds (Sum of triceps and subscapular)	− 0.69	−2.33, 0.95	0.410	− 0.94	−2.82, 0.93	0.324
**Physical Fitness**						
Standing long jump (cm)	−1.18	−4.44, 2.07	0.476	− 0.99	− 4.71, 2.73	0.602
Handgrip strength (kg)	0.78	0.08, 1.48	**0.028**	0.81	0.02, 1.61	**0.046**
20-m shuttle run test (CRF) (VO_2max_ ml/kg/min)	0.56	−0.42, 1.54	0.262	0.74	−0.38, 1.85	0.193

Abbreviations: *AST* Active School Transport, *PA* Physical Activity, *LPA* Light Physical Activity, *MVPA* Moderate-to-Vigorous Physical Activity, *BMI* Body Mass Index, *CRF* Cardiorespiratory fitness, *VO*_*2max*_ Maximum Oxygen Consumption, *95%CI* 95% Confidence Intervals; (β), Regression Coefficient

*All analyses were adjusted by sex, age and socioeconomic status; robust standard errors were used to account for potential clustering within the 23 primary schools and the 18 secondary schools

Results from the path analysis are presented in Table 3. Path analyses showed that (1) AST volume at baseline had no total effect on physical fitness and adiposity indicators at T2 (all, *p* > 0.05) (path *c* ’); (2) AST volume at baseline had a positive effect on MVPA at T1 (all, β = 0.33; *p* < 0.001), but not on LPA at T1 (all, β = 0.20; *p* > 0.05) (path *a*); (3) MVPA at T1 mediated associations between AST at T0 and CRF at T2 (β = 0.21; *p* = 0.037) (path *b*); and (4) no indirect effects among AST volume at baseline on physical fitness and adiposity indicators at T2 via LPA and MVPA at T1 (potential mediators) were identified (all, *p* > 0.05) (product of paths *a* and *b)*.

**Table 3:** Longitudinal mediation effect of PA (T1) between AST volume (T0) and adiposity and fitness (T2)

Independent Variable	Potential mediator	Outcomes	a-path β (95%CI)	b-path β (95%CI)	Total Effect c-path β (95%CI)	Direct Effect c’-path β (95%CI)	Indirect Effect a^*^b β (95%CI)
AST volume	LPA	BMI	0.20 (− 0.04;0.43)	−0.02 (− 0.04;0.00)	0.03 (− 0.05;0.11)	0.04 (− 0.04;0.12)	−0.00 (− 0.01;0.00)
		Skinfolds	0.20 (−0.05;0.45)	0.00 (−0.12;0.13)	− 0.06 (− 0.48;0.36)	−0.07 (− 0.48;0.35)	0.00 (− 0.02;0.03)
		Waist circumference	0.20 (−0.05;0.44)	− 0.04 (− 0.12;0.04)	0.04 (− 0.28;0.37)	0.05 (− 0.28;0.38)	−0.01 (− 0.03;0.01)
		Standing long jump	0.20 (−0.05;0.44)	−0.02 (− 0.44;0.40)	−0.37 (− 1.75;1.01)	−0.37 (− 1.73;1.00)	−0.00 (− 0.08;0.08)
		Handgrip strength	0.20 (−0.06;0.45)	−0.01 (− 0.07;0.05)	−0.12 (− 0.36;0.13)	−0.11 (− 0.36;0.13)	−0.00 (− 0.01;0.01)
		20-m shuttle run test	0.20 (−0.04;0.44)	0.01 (−0.09;0.12)	0.09 (− 0.26;0.44)	0.09 (− 0.26;0.44)	0.00 (− 0.02;0.02)
	MVPA	BMI	**0.33 (0.17;0.50)**	−0.04 (− 0.08;0.01)	0.04 (− 0.03;0.12)	0.06 (− 0.03;0.14)	−0.01 (− 0.03;0.00)
		Skinfolds	**0.33 (0.16;0.49)**	−0.05 (− 0.22;0.12)	−0.05 (− 0.47;0.37)	−0.04 (− 0.46;0.39)	−0.02 (− 0.07;0.04)
		Waist circumference	**0.33 (0.18;0.49)**	−0.04 (− 0.21;0.13)	0.07 (− 0.26;0.41)	0.09 (− 0.25;0.42)	−0.01 (− 0.07;0.04)
		Standing long jump	**0.33 (0.18;0.49)**	0.22 (−0.34;0.77)	−0.47 (− 1.85;0.91)	−0.54 (− 2.03;0.95)	0.07 (− 0.11;0.26)
		Handgrip strength	**0.33 (0.17;0.49)**	−0.06 (− 0.18;0.06)	−0.11 (− 0.35;0.14)	−0.09 (− 0.33;0.16)	−0.02 (− 0.06;0.03)
		20-m shuttle run test	**0.33 (0.18;0.50)**	**0.21 (0.01;0.40)**	−0.01 (− 0.34;0.37)	−0.06 (− 0.42;0.31)	0.07 (− 0.01;0.15)

Abbreviations: *PA* Physical Activity, *T0* Baseline, *T1* 1-year follow-up, *T2* 2-year follow-up, *AST* Active School Transport, *LPA* Light Physical Activity, *MVPA* Moderate-to-Vigorous Physical Activity, *BMI* Body Mass Index, *VO*_*2max*_ Maximum Oxygen Consumption, *AST* Active School Transport; Path *a*, the effect of the AST at T0 (exposure) on PA at T1 (potential mediator), adjusted for T0 level of PA; Path *b*, the effect of PA at T1 on the outcome at T2, adjusted for level of the outcome at T1; Path *c,* the total effect of T0 AST on the outcome at T2, adjusted for T0 and T1 levels of both the outcome and PA; Path *c* ’*,* the direct effect of T0 AST on the outcome at T2, adjusted for T1 levels of both the outcome and PA; Path a^*^b, the indirect effect of T0 AST on the outcome at T2, adjusted for T0 levels of both the outcome and PA; 95%CI, 95% Confidence Intervals; (β), Regression Coefficient

^*^All analyses were adjusted by sex, age and socioeconomic status; robust standard errors were used to account for potential clustering within the 23 primary schools and the 18 secondary schools

In addition, we repeated all the analyses after adjusting for living in urban or rural areas and total wear time, and the results did not change (data not shown).

## Discussion

The current study provides novel and valuable information about the longitudinal associations of AST, PA levels, physical fitness and adiposity indicators over three time-points in children and adolescents from Cádiz and Madrid, respectively. We found significant declines in time spent in LPA and MVPA levels across the three time-points and increases in handgrip strength. The mediation analysis suggested that the positive association between AST volume and CRF over time was partially mediated by MVPA in children and adolescents.

Overall, the volume of AST was stable among participating children and adolescents over the 2-year follow-up compared with other longitudinal studies. In Australia and Belgium, for example, frequency and duration of AST have been shown to increase over time in children and adolescents [43–45]. The longitudinal studies performed by De Meester et al. [45] and by Cardon et al. [43] in Belgium included two and five time-points of measurements, respectively, however unlike the current study they examined change in AST over the transition from primary to secondary school. Consequently, the transition from primary to secondary school may explain this increment in AST volume, whereby the distance between home and school, neighbourhoods and facilities from secondary schools may facilitate more time in AST than the travel to primary schools [44, 45]. It is also possible that those attending secondary schools may be afforded more independence and rely less on parents to transport them to school [46]. Another explanation may be that adolescents are more likely to be active commuters to school than children [47–49], as parents place fewer restrictions as they are more confident of their ability to negotiate traffic safely [50].

Participants in the current study spent an average of 76 min/week (or 15.2 min/day) in AST at baseline. This is slightly higher than time reported in other longitudinal and cross-sectional studies with similar aged (10 to 13 years old) samples; 11.6 min/day [51], 11.4 min/day [45], and 8.5 min/day [52]. Thus, although the decrease in LPA and MVPA seen in this study is consistent with many others [53], the stability of AST is promising as it appears that AST remains a consistent and habitual source of PA over time in this sample. This is important as our findings also show that AST volume at T0 was significantly positively associated with MVPA 1 year later (T1) in the path analyses. In addition, the classification observed in the change of AST in the ratio active/passive (*n* = 13, 2.9%) and passive/active (*n* = 25, 5.6%) commuters from T0 to T2, respectively, confirms that the sample was constant over time with a 2.7% of the participants who joined to the AST behaviour. These results are in agreement with previous cross-sectional studies [52, 54–57], where children and adolescents, who actively commute to school, were shown to be more likely to meet MVPA guidelines.

Additionally, the longitudinal path analysis showed that the relationship between AST volume and CRF was indirectly explained (or mediated) by changes in MVPA. It is not surprising that there was a relationship between AST and CRF mediated by MVPA, and not the other components of physical fitness measured, upper body (handgrip) or lower body strength (standing long jump), where AST is less likely to have an impact on these measures and the scarce literature regarding muscular fitness is inconclusive [22, 58]. A systematic review observed that children who bicycled to school showed higher values of CRF when compared to passive commuters, with little evidence for associations between walking to school and CRF [59]. Moreover, Larouche et al. reported that evidence is also scarce and inconclusive due to the variability of the study designs and CRF measures [20]. However, Voss et al. [60] showed that children who walk to school had higher CRF, measured by 20 m shuttle run test, compared to passive commuters in a large sample (*n* = 6085, aged 10.0–15.9 years old). Our longitudinal results in a sample where most AST was via walking suggests protective effects of AST over time, especially in the context of declining LPA and MVPA with age. Thus, AST may be an important source of PA that helps to preserve physical fitness in the context of age-related declines in overall MVPA. However, we acknowledge that AST is likely to make only a partial contribution to overall MVPA. Consequently, although CRF is a result of being active in other forms of PA, the amount of AST would contribute as a fraction of the overall MVPA and, consequently, reach LPA levels. Moreover, the non-significant relation between AST and CRF could be due to the stable AST behavior along the longitudinal study, since the sample was constant over time. Investment in efforts to promote and preserve the practice of AST in children and adolescents is therefore critical as AST may be a low cost and effective [59] tool to increase PA levels and health-related physical fitness in youth.

Finally, the longitudinal path analysis showed that the non-relationship between AST volume and adiposity indicators was not clarified by changes in LPA or MVPA. Some systematic reviews have shown inconsistent findings between the AST and adiposity indicators among youth [20, 55]. These discrepancies could be explained by the fact that most studies combined walking and cycling to school in their analyses [20, 55] or the country differences in cycling and walking habits [61]. Thus, in countries such as Spain or Greece, where cycling to school is uncommon, the overweight rates are higher than in countries with high cycling frequencies (e.g. Norway or the Netherlands), where the rates are lower [61]. In the present study, where the majority of the participants were walkers (*n* = 445), could indicate that walking to school does not have enough potential to improve body adiposity as has been described in other studies [22, 62, 63]. Therefore, it is not unexpected that in countries where cycling is more common, AST is associated with less adiposity [63–65].

### Strengths and limitations

The longitudinal design and path and multilevel analyses are clear strengths, as is the consideration of a range of outcomes, including physical fitness (e.g., CRF, strength, and power), and indicators of adiposity and the novel mediation hypothesis. However, a large proportion of the sample was lost due to the need to restrict analyses to complete cases for the path analyses. This may have introduced some bias, as a higher percentage of those included in analyses spent less time in AST and MVPA, had higher SES and were from urban areas than those participants who were excluded. The results are therefore likely to be underestimated. The sample size is not representative of the Spanish youth, therefore, caution should be taken with the generalizability of the results. In addition, the small sample size also prohibited stratification of the sample according to age or sex. The use of self-reported measures of AST volume might be associated with less precision and accuracy than other measurement approaches. Finally, ‘usual’ AST volume was reported in this study and may not reflect LPA and MVPA measured by accelerometry if the 7 days monitored did not reflect habitual PA.

## Conclusions

While PA declined over time, AST remained stable. Results from the longitudinal path analysis suggest that AST volume may be an important source of PA that helps to preserve CRF in the context of age-related declines in overall MVPA among Spanish children and adolescents. Therefore, we encourage health authorities to promote AST as an important means of gaining PA and enhancing health-related physical fitness in youth. Further longitudinal studies are needed to identify determinants of AST over time to inform future intervention strategies.

## Supplementary information

**Additional file 1: Table S1.** Changes over time in AST volume, PA, adiposity and fitness for whole sample (*n* = 1646).

## Data Availability

The datasets used and/or analysed during the current study are available from the corresponding author on reasonable request.

## Abbreviations

AST: Active school transport
BMI: Body mass index
CPM: Counts per minute
CRF: Cardiorespiratory fitness
FAS: Family affluence scale
LPA: Light physical activity
MVPA: Moderate- to vigorous-intensity physical activity
PA: Physical activity
SES: Socioeconomic status
T0: Baseline
T1: 1-year follow-up
T2: 2-year follow-up
