# Supplementary material for: Changes in and the mediating role of physical activity in relation to active school transport, fitness and adiposity among Spanish youth: the UP&DOWN longitudinal study
Source: Int J Behav Nutr Phys Act. 2020 Mar 10;17:37. doi: 10.1186/s12966-020-00940-9 (PMC7063792; doi:10.1186/s12966-020-00940-9)
Supplement: Supplementary file 1 — Additional file 1: Table S1. Changes over time in AST volume, PA, adiposity and fitness for whole sample (n = 1646). [file 12966_2020_940_MOESM1_ESM.docx]

**Table S1.** Changes over time in AST volume, PA, adiposity and fitness for whole sample (n=1646).

|  | **1-year follow-up** | | | **2-year follow-up** | | |
| --- | --- | --- | --- | --- | --- | --- |
|  | **β** | **95%CI** | ***P* value** | **β** | **95%CI** | ***P* value** |
| **Mode of commuting** |  |  |  |  |  |  |
| AST volume (min/week) | 5.79 | -2.12 , 13.70 | 0.151 | 5.30 | -3.56 , 14.16 | 0.241 |
| **Physical Activity Levels** |  |  |  |  |  |  |
| LPA (min/day) | -10.16 | -12.99 , -7.33 | **<0.001** | -14.81 | -18.06 , -11.56 | **<0.001** |
| MVPA (min/day) | -3.30 | -5.13 , -1.46 | **<0.001** | -3.79 | -5.90 , -1.68 | **<0.001** |
| **Adiposity indicators** |  |  |  |  |  |  |
| BMI (kg/cm^2^) | 0.12 | -0.14 , 0.38 | 0.376 | -0.08 | -0.38 , 0.22 | 0.592 |
| Skinfolds (Sum of triceps and subscapular) | -0.18 | -1.17 , 0.81 | 0.725 | -0.54 | -1.69 , 0.61 | 0.356 |
| **Physical Fitness** |  |  |  |  |  |  |
| Standing long jump (cm) | -1.03 | -2.99 , 0.93 | 0.304 | -2.94 | -5.20 , -0.68 | **0.011** |
| Handgrip strength (kg) | 0.47 | 0.09 , 0.86 | **0.016** | 0.74 | 0.29 , 1.18 | **0.001** |
| 20-m shuttle run test (CRF) (VO_2max_ mL/kg/min) | 0.36 | -0.16 , 0.88 | 0.170 | 0.58 | -0.02 , 1.18 | 0.059 |

Abbreviations: AST, Active School Transport; PA, Physical Activity; LPA, Light Physical Activity; MVPA, Moderate-to-Vigorous Physical Activity; BMI, Body Mass Index; CRF, cardiorespiratory fitness; VO_2max,_ Maximum Oxygen Consumption; 95%CI, 95% Confidence Intervals; (β), Regression Coefficient.

*All analyses were adjusted by sex, age and socioeconomic status; robust standard errors were used to account for potential clustering within the 23 primary schools and the 18 secondary schools.
